# Successful Intraosseous Thrombolysis in the Management of a Massive Pulmonary Embolism With Cardiac Arrest

**DOI:** 10.7759/cureus.12105

**Published:** 2020-12-16

**Authors:** Kirsty Nweze, Clarissa S Ribeiro, James Kelly, Joaquim Cevallos Morales

**Affiliations:** 1 Intensive Care Unit, Newham University Hospital, London, GBR; 2 Intensive Care Unit, Newham University Hospital / Barts NHS Health Trust, London, GBR

**Keywords:** emergency medicine, haemodynamic monitoring, resuscitation, shock, medical intensive care unit (micu)

## Abstract

We describe the successful cardiopulmonary resuscitation of a patient with massive pulmonary embolism who received thrombolysis via the intraosseous route. This case also demonstrates survival without apparent long-term sequelae despite extreme metabolic acidosis. In the context of pulmonary embolism, this has not been widely reported in the existing literature. A 22-year-old woman suffered a prolonged cardiac arrest secondary to pulmonary embolism in a hospital corridor following short hospital admission for medical termination of pregnancy. A point-of-care echocardiogram showed a grossly dilated right ventricle indicative of pulmonary embolism. Due to severe peripheral vasoconstriction, intravenous access proved difficult, and the decision was made to deliver intraosseous thrombolysis. Initial blood gas analysis showed a profound acidosis due to alternating return of spontaneous circulation and further loss of output. Because of her prolonged "low-flow" state, she was deemed unsuitable for extracorporeal membrane oxygenation. Despite the poor prognosis, the decision was made to continue with resuscitation in light of a reversible pathology. She was successfully discharged from the hospital after a short intensive care stay with no long-term complications. This case demonstrates successful thrombolysis through an intraosseous route, with a good outcome despite poor prognostic factors. Early thrombolysis and continuous cardiopulmonary resuscitation in massive pulmonary embolism are imperative to survival in cardiac arrest.

## Introduction

Thrombotic events are common in obstetric patients, occurring up to ten times more frequently than in the general population. Acute pulmonary embolism (PE) can be rapidly fatal in cardiac decompensation cases and is one of the leading causes of maternal deaths in developed countries [1]. Here, we present a case of massive PE leading to cardiac arrest in a patient following medical termination of pregnancy. She underwent successful thrombolysis during cardiopulmonary resuscitation (CPR) and, despite 13 cycles of CPR and over an hour in a “low-flow” state, survived to discharge with no evidence of neurological impairment.

## Case presentation

A 22-year-old woman of nine weeks gestation was admitted to the gynecology ward for medical termination of pregnancy. She was discharged two hours following the procedure. A clinical review prior to discharge noted no evidence of hemodynamic instability and normal vital signs. The patient was advised to restart treatment-dose low molecular weight heparin (tinzaparin) for a left lower lobe PE diagnosed two weeks previously. Of note, past medical history included a previous PE in pregnancy one year ago, treated with tinzaparin, and two previous miscarriages, which had not been referred for further investigation.

Following discharge, the patient collapsed in a hospital corridor, and a cardiac arrest call was put out. On the arrival of the arrest team, the patient was found to be extremely agitated, with a fluctuating level of consciousness (Glasgow Coma Score 13; E3 V4 M6). Clinical signs revealed central cyanosis, a delayed capillary refill time of five seconds, and marked sinus tachycardia. Brachial blood pressure was unrecordable, and oxygen saturations read at 80%. Intravenous (IV) access was extremely difficult to secure due to marked peripheral vasoconstriction. After approximately five minutes, there was a loss of cardiac output, and CPR was commenced. Initial and subsequent rhythms were of pulseless electrical activity (PEA). Intraosseous access was secured, and subsequent venous access was acquired via the external jugular vein. A supraglottic airway device was inserted to maximize oxygenation. The advanced life support algorithm was followed, with adrenaline given at alternate cycles, and return of spontaneous circulation (ROSC) was achieved four times. An echocardiogram showed a grossly dilated right ventricle, consistent with massive PE. Thrombolysis was administered via the intraosseous line with alteplase (recombinant tissue plasminogen activator [r-tPA]) 50mg IV bolus, 34 minutes after the initiation of CPR as per Trust guidelines. Definitive ROSC occurred after 13 cycles of CPR, with a total “low-flow” time (defined as the interval between initiation of CPR and ROSC), of 66 minutes. The last venous blood gas showed a profoundly mixed acidosis (pH 6.56, the partial pressure of carbon dioxide [pCO_2_] 17, potassium [K+] 3.8, lactate: 24, bicarbonate [HCO_3_] 4, hemoglobin [Hb] 117). 

The patient was transferred to the intensive care unit, where she was intubated with a modified rapid sequence induction with ketamine. A further 40mg of IV alteplase over two hours was given as per the manufacturer's protocol. Following discussion with hematology, a therapeutic dose of tinzaparin was also administered. Continuous lithium dilution cardiac output (LiDCO) monitoring was initiated. Parameters were in keeping with cardiogenic shock (cardiac index 2.5 L/min/m2, cardiac output 5.0 L/min, stroke volume variation 14%, systemic vascular resistance 1,562 dynes•sec/cm^2^), requiring a maximum dose of dobutamine of 10 micrograms/kg/min, which was combined with vasopressin and noradrenaline to ensure mean arterial pressures of 65 and above. An urgent referral to a tertiary center for extracorporeal membrane oxygenation (ECMO) was made for cardiac and respiratory support but was declined on the grounds of the prolonged duration of a low-flow state, and it was advised that the case was managed locally. 

**Figure 1:**
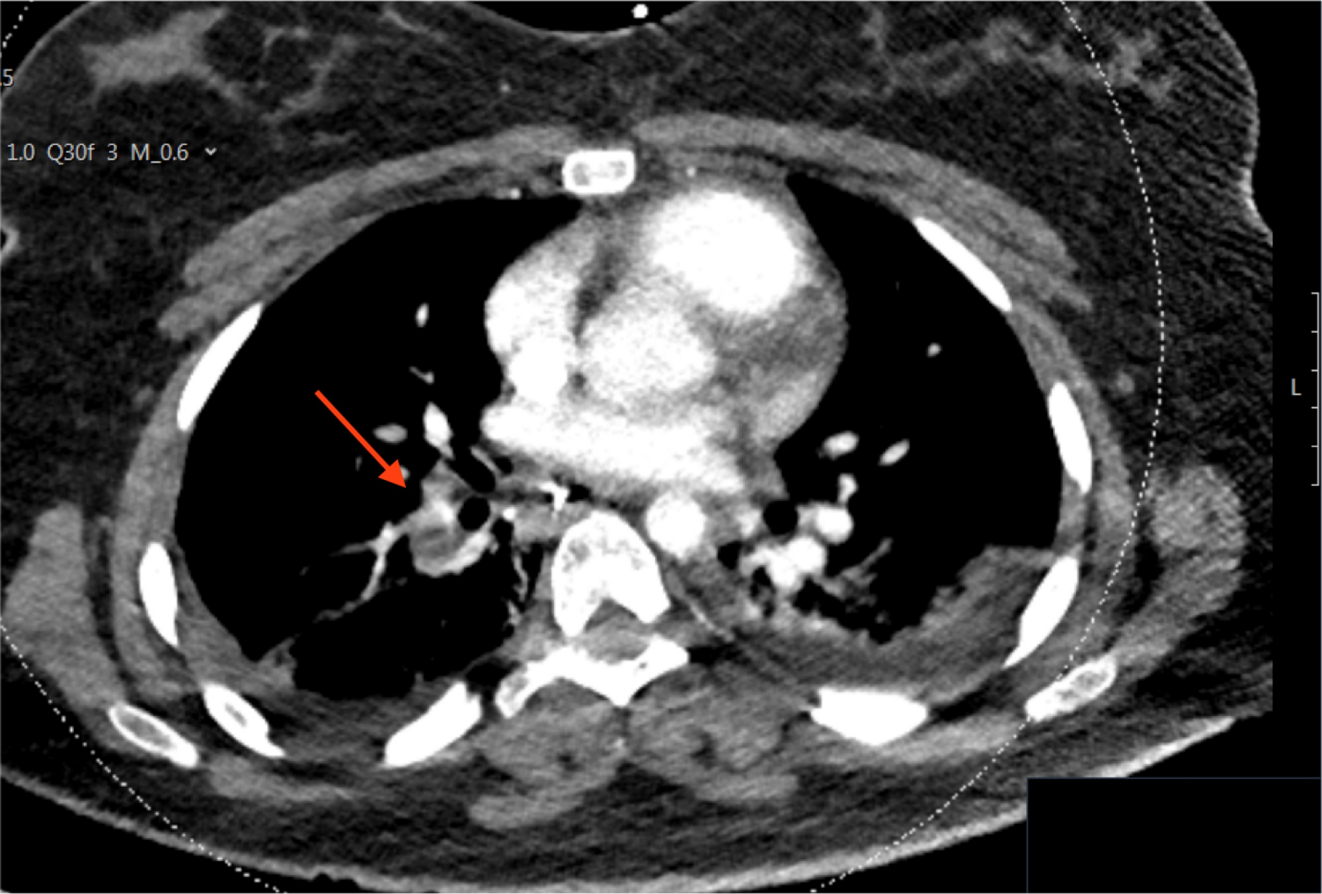
CTPA demonstrating a filling defect of the right pulmonary artery CTPA - computerized tomography pulmonary angiogram

The following day, a computerized tomography pulmonary angiogram (CTPA) showed new pulmonary emboli on the right side (Figure 1).

An early formal bedside echocardiogram showed an ejection fraction of 50% with a grossly dilated right ventricle, impaired systolic function, and mild tricuspid regurgitation. Pulmonary pressures could not be recorded. Subsequently, the patient developed a stage 3 acute kidney injury with urine output maintained at 0.5 ml/kg/hr and worsening liver function tests (international normalized ratio [INR] 4.5, alanine aminotransferase [ALT] 1,174, alkaline phosphatase [ALP] 84, albumin 30, bilirubin 18), in keeping with a secondary ischaemic organ injury. Consequently, anticoagulation was held for two days. Factor Xa levels were persistently low. The patient slowly improved and was extubated after two days. Vasopressor and inotrope support were also successfully weaned, and the ischaemic liver and kidney injury started to resolve. She was stepped down to the high dependency unit and discharged from the hospital after 14 days. 

## Discussion

Epidemiological studies report a global incidence of PE in 39 to 115 individuals per 100,000 population [2]. Several countries have reported that the incidence has had an increasing trend over the past two decades. This may be due to increasing numbers of patients surviving severe comorbidities, such as malignancy, which carry a higher risk of developing venous thromboembolism (VTE). It may also be attributable to the availability of more accurate diagnostic imaging and a lower threshold of suspicion for the disease. Amongst women aged 15-55 years old in Europe, the number of deaths attributed to acute PE has been reported to be between 8-13 per 1,000 deaths, despite the overall downward European trend in mortality rates since the year 2000 [1]. Even with advances in imaging and antiplatelet therapy, acute PE still remains one of the leading causes of mortality in Western countries and is the second most common cause of maternal deaths in the UK, accounting for 16% [3]. In addition, the risk of VTE is higher during the postpartum period than during pregnancy; thus, vigilant monitoring for symptoms during this time is preeminent. A past medical history of thrombosis is the most important risk factor for VTE, increasing the risk of recurrent VTE three to fourfold (relative risk: 3.5%; 95% CI: 1.6-7.8); thus, pregnant women with a previous history of VTE require frequent and cautious monitoring due to the compounded higher risk of morbidity. The presence of thrombophilia is the next most important risk factor for VTE, with both acquired and inherited conditions increasing the risk [4]. According to guidance from the Royal College of Obstetricians and Gynaecologists, pregnant women with recurrent VTE should be managed in a joint obstetric and hematology clinic throughout the antenatal and peripartum period, with input from clinicians experienced in managing thrombophilic conditions. Such patients are usually offered thromboprophylaxis following categorization into lower and higher risk groups. An underlying hypercoagulable condition found will likely have a high impact on future decisions regarding subsequent pregnancies and choice of long term prophylaxis [5].

In this particular case, the indications for thrombolysis were complete hemodynamic collapse and echocardiographic evidence of right ventricular dilatation and strain on a background of previous PE and the pro-thrombotic state of pregnancy. Although a normal echocardiogram does not preclude the diagnosis of PE, the presence of severe hemodynamic instability and convincing evidence of right ventricular dysfunction are deemed reliable enough to diagnose a massive PE and thus are recognized as criteria for thrombolysis in the British Thoracic Society (BTS) guidelines [6, 7]. Additionally, echocardiography has been shown to prognosticate in acute PE, with a right ventricular hypokinesis recognized as an independent risk factor for increased 30-day mortality in patients with systolic blood pressures above 90 mmHg (hazard ratio: 1.94; 95% CI: 1.23-3.06) [8]. However, some studies have suggested that right ventricular dysfunction is a more accurate predictor of mortality in hemodynamically unstable patients [9].

Several studies have demonstrated ROSC following thrombolysis in PEA cardiac arrest secondary to confirmed massive PE. In particular, the PEAPETT (pulseless electrical activity in pulmonary embolism treated with thrombolysis) trial reported ROSC within 15 minutes of alteplase administration in 22 out of 23 patients with massive PE, with an average time to administration of 6.5 mins [10]. As in our case, alteplase was administered as a 50mg IV bolus over one minute. However, due to the complexity of managing an arrest in a corridor, moving a hemodynamically unstable patient, and difficulty locating alteplase in our hospital, thrombolysis was not delivered until 34 minutes following the cardiac arrest initiation call.

This case illustrates that the complexity of managing critically unwell patients outside of the intensive care unit can be mitigated by good quality, uninterrupted CPR to increase forward blood flow and maintain adequate perfusion to end-organs. In addition, our patient’s physiological reserve and bystander CPR were also likely important factors in her successful outcome. 

Additional case reports illustrate that ROSC can be achieved in PEA cardiac arrests secondary to PE in post-operative patients, patients with organ failure, following periods of immobility or spontaneous PEs, following administration of tissue plasminogen activator at varying bolus doses [11]. We opted for 50 mg IV alteplase as per BTS guidelines, followed by a 40 mg IV infusion over two hours (total dose 90 mg) as per manufacturer guidelines. It is unclear whether there is any benefit of bolus dosing over infusion, although some suggest the use of dynamic echocardiographic monitoring to guide alteplase dosing in massive PE, and this remains an interesting area of future research [12, 13].

Given the difficulty in securing access in this patient, the decision was made to thrombolyse via an intraosseous (IO) line inserted into the proximal left tibia, which was secured rapidly prior to cannulation of the external jugular vein. This case lends further weight to suggesting that the administration of thrombolysis during cardiac arrest via the IO route is safe where venous access is difficult [14, 15]. Whilst there are potential risks of inducing hemorrhage, air emboli, fractures, and tissue necrosis [16], it was felt that the potential benefits outweighed the risks of administration in this critically unwell patient. She did not suffer any bleeding or neurovascular complications as a result.

The total duration of the “low-flow” state was 66 minutes, and the final venous blood gas for the patient demonstrated extreme mixed acidosis (pH 6.56, lactate 24, pCO_2_ 17), indicative of profound organ hypoperfusion. Indeed, acidosis is a cardiotoxic state and can impair oxygen delivery to tissues, making resuscitation attempts even more difficult. Cases of ROSC with full recovery in patients with intra-arrest acidosis below this have been documented in hemorrhagic shock, raised anion-gap acidosis, and drowning, but not following PE-induced cardiac arrest [17]. This raises the ethical debate as to when to cease resuscitation efforts in cases of potentially reversible cardiac arrest causes and what defines the "point of futility". During and after resuscitation, attempts were made to urgently refer the patient to a tertiary cardiac center for ECMO, but the referral was declined because it was felt that the persistent hypoperfusion meant that ECMO was unlikely to be successful. Whilst this may have informed a decision to stop resuscitation, it was felt that all efforts should be made to revive and intensively treat the patient in light of a reversible pathology. She made a full neurological recovery and is being followed up in a thrombophilia clinic. 

## Conclusions

Pulmonary embolism is a condition that has increased in incidence over the last few decades, in large part due to modern risk factors, including chronic disease, obesity, and malignancy. However, with advances in diagnostic imaging, earlier treatment has improved outcomes. This case demonstrated the successful cardiopulmonary resuscitation and intraosseous thrombolysis of a young female and survival to discharge without evident long term sequelae. This successful outcome can be attributed to high suspicion of PE, an unconventional route of thrombolysis, and persistence in resuscitation despite the profound metabolic acidosis.
